# Impact of a Postoperative Intervention Educational Program on the Quality of Life of Patients with Hip Fracture: A Randomized, Open-Label Controlled Trial

**DOI:** 10.3390/ijerph17249327

**Published:** 2020-12-13

**Authors:** Francisco Javier Amarilla-Donoso, Raúl Roncero-Martín, Jesus Lavado-García, María de la Luz Canal-Macías, María Pedrera-Canal, Carlos Chimpén-López, Rosaura Toribio-Felipe, Sergio Rico-Martin, Sabina Barrios-Fernández, Fidel López-Espuela

**Affiliations:** 1 Department of Nursing, Hospital Campo Arañuelo, Calle Tomas Yuste S/N, Navalmoral de la Mata, 10300 Cáceres, Spain; javier.amarilla@gmail.com; 2 Metabolic Bone Diseases Research Group, Nursing Department, Nursing and Occupational Therapy College, University of Extremadura, Avd. Universidad s/n, 10003 Cáceres, Spain; rronmar@unex.es (R.R.-M.); luzcanal@unex.es (M.d.l.L.C.-M.); sergiorico@unex.es (S.R.-M.); fidellopez@unex.es (F.L.-E.); 3 Department of Nuclear Medicine, Hospital Clínico San Carlos, Av. Profesor Martín Lagos s/n., 28040 Madrid, Spain; mariapedreracanal@gmail.com; 4 Nursing and Occupational Therapy College, University of Extremadura, Avd. Universidad s/n, 10003 Cáceres, Spain; cchimpen@unex.es (C.C.-L.); sabinabarrios@unex.es (S.B.-F.); 5 Department of Nursing, Hospital Virgen del Puerto, Carretera del Puerto s/n, 10600 Cáceres, Spain; rotofe@gmail.com

**Keywords:** quality of life, hip fracture, educational program, geriatric care, old people

## Abstract

The objective of this study was to determine the impact of a postoperative educational intervention program on the health-related quality of life (HRQoL) of patients with hip fracture using a controlled clinical trial in a randomized, multicenter study. In total, 102 patients (45.5%) from trauma units at the two University Hospitals of the province of Cáceres received the educational program, whereas 122 (54.5%) did not. Patients were consecutively included in either an intervention or a control group. Patients from the intervention group received an educational program during admission and the postoperative period. Patients from the control group did not receive any educational program. These patients were managed according to routine protocols. The patients were predominantly female (76.3%), aged 84.6 years (SD 6.1). All dimensions in both groups at 12 months showed a significant decrease with respect to baseline, except for bodily pain in both groups (*p* = 0.447; *p* = 0.827) and social functioning in the intervention group (*p* = 0.268). Patients receiving the educational program showed higher levels in the dimensions of the Mental Component Summary (MCS-12) (*p* = 0.043), vitality (*p* = 0.010), and social functioning (*p* < 0.001), as well as in the dimensions of the SF-12 health survey questionnaire of HRQoL 12 months after surgery. In conclusion, our study of the intervention group showed that there were significant improvements in MCS-12, vitality, and social function dimensions compared to the control group.

## 1. Introduction

Hip fracture is one of the most important social and health problems, due to its increasing prevalence, health consequences, and economic impact [1]. In Spain, the incidence of hip fractures is around 104 cases per 100,000 inhabitants, which represents between 45,000 and 50,000 hip fractures per year, of which 76% occur in women [2].

Hip fractures generally occur in people over 65 mostly associated with a fall, and their number increases significantly with age; almost half of hip fractures occur in the elderly (85 years and older) [3]. The fundamental cause of this association is the increase in risk factors for falls in the elderly population, added to a reduction in bone quality associated with age [4]. The increase in life expectancy will significantly affect the number of hip fractures; according to the World Health Organization, the number of hip fractures associated with osteoporosis will triple in the next 50 years, going from 1.7 million cases in 1990 to 6.3 in 2050 worldwide [5].

This condition is also associated with significant morbidity and mortality. After a hip fracture, physical and psychological limitations are common, such as reduced mobility, impaired balance, lack of confidence, or fear of falling [6,7]. Between 25% and 75% of people who walked independently before the fracture become dependent after 1 year or do not reach the same level before the fracture [8]. Older hip fracture patients are at high risk for psychological problems related to the traumatic nature of the injury. The mortality rate is 8.86 (8.79–8.93) per 1000 people/year [9], representing a loss of 7.218 quality-adjusted life years [2].

The annual cost of treating hip fractures is around EUR 1.591 million, which represents more than 2.5% of total healthcare spending [2]. The costs are derived from expensive hospitalizations and long rehabilitation processes, in addition to the indirect costs associated with the patient’s environment as a result of the change in lifestyle.

All these aspects have a significant negative impact on the health-related quality of life (HRQoL) of patients [10,11], which can also be maintained for long periods of time despite successful treatment [12,13]. Vertebral and hip fractures have a considerably greater and longer impact on HRQoL than other fractures [14].

Early initiation of a personalized care plan aimed at restoring motion and function and minimizing the risk of another fracture should be among the goals of clinical care for hip fracture patients.

After the surgical treatment of a hip fracture, patient-reported outcome tools are required to assess not only the impact, but also the efficacy of all medical and surgical interventions on patients’ perceived health status and HRQoL. Progressively, the latest research has shown the increasing importance of obtaining a representation of the state of health in a multidimensional way through the integration of indicators that take into account the needs of patients and their families. HRQoL has become a very important variable to decide the therapeutic path, since it is not only enough to avoid morbidity and mortality of a certain pathology; it is also essential to maintain or improve HRQoL [15,16].

Educational programs aimed at improving patient self-care and self-efficacy have been shown to improve patient functionality and adherence to recommendations [17,18].

The main objective of this study was to compare an educational program focused on self-efficacy and developed during the immediate postoperative period against standard clinical care. The primary outcome to be compared between the groups was HRQoL at baseline and at 12 months after surgery.

## 2. Methods

### 2.1. Study Design

A controlled clinical trial (NCT04650360) was applied, randomized with two parallel groups of patients recruited from the trauma and orthopedic units of two university hospitals in the province of Cáceres, during the period of June 2015 to July 2017.

We aimed to have enough statistical power to detect medium effect sizes (anticipated Cohen’s d = 0.4) with a β = 0.80 and α = 0.05, which required a minimum sample size of 200 participants [19]. A total of 224 hip fracture patients (aged 84.6 (±6.1) years) were included in this study.

The selected patients were randomly allocated to either the trial group or control group using computer-based randomization.

The inclusion criteria were patients over 65 years of age admitted with a primary diagnosis of hip fracture, who underwent urgent surgical intervention for surgical fixation of the fracture, who were without cognitive impairment, who were not in a terminal situation, and who did not present language barriers that made it difficult to understand the educational program or the different questionnaires.

With the exception of the intervention under study, all patients included in the study were treated according to the usual clinical practice. Written informed consent was obtained to participate in the study. The procedures were adjusted to the provisions of the Declaration of Helsinki and were approved by the Clinical and Ethical Research Committee (Ref.15/0145) of Cáceres (Spain). At the time of hospital admission, the patients were consecutively included in either an intervention or a control group.

### 2.2. Intervention

Patients assigned to the intervention group received an educational program during admission. The health educational program consisted of a single training session offered by a nursing professional to each patient and caregiver. In each educational session, the following topics were addressed: objectives for functional recovery (early mobilization, recovery of functional capacity lost prior to the fracture, etc.), mobilization exercises to start the day after the surgical procedure (lower limb exercise, respiratory physiotherapy, etc.), and tips to prevent future falls. The educational session was implemented during the postoperative hospital stay, with an approximate duration of 30–45 min. The session ended with a summary of the content and comments from the patient and relative in order to ensure understanding of the program. Written information was provided on the aspects addressed in the session (brochures). The patients in the control group did not receive any educational program. These patients were treated according to routine protocols. All patients were monitored during admission, at 1 month, at 6 months, and at 1 year after the intervention.

### 2.3. Variables

For data collection, a questionnaire was developed that contained the following variables: clinical, sociodemographic, and economic data, personal history (e.g., prior diagnosis of osteoporosis, defined as bone mineral density T-score hip or lumbar spine less than or equal to -2.5 SD- [20]), usual treatment, clinical variables of functional dependence (Barthel index, Lawton and Brody scale), socio-family assessment (Gijon scale), health-related quality-of-life variables (SF-12 health survey), days of hospital stay, delay in surgical intervention, destination after discharge, functional ambulation capacity, and presence of complications, falls, and polymedication (five or more medications daily for a period of more than six months).

The Charlson comorbidity index (CCI) was used as a method of quantifying the number of chronic disorders together with their severity. The American Society of Anesthesiologists (ASA) scale was used to classify the physical state. This questionnaire was completed during the time of admission and 1 month after hospital discharge, at the follow-up consultation. The data were obtained through a personal interview with the patient and a review of the medical records of the hospitalization process. In order to know the baseline situation, the participants were interviewed about their functional situation and quality of life by referring to two weeks before the fracture, identifying these data as the baseline situation.

For the quantification of HRQoL, the SF-12 Health Survey was used, which consists of two dimensions (Physical Component Summary (PCS-12) and Mental Component Summary (MCS-12)) that measure eight health domains (PCS: general health, physical function, physical role, and body pain; MCS: social function emotional role, mental health, and vitality) divided into a summary score of the physical component [21,22]. Each component can be scored from 0 (lowest health) to 100 (highest health).

The ability to perform basic activities of daily living (ABVD) was evaluated using the Barhtel Index [23]. This scale evaluates 10 elements (food, bathroom, clothing, toilet, bowel movements, urination, use of toilet, transfers, mobility, and stairs). A total score between 0 and 20 suggests total dependence for the development of BALS, a total score between 21 and 60 suggests severe dependence, a total score between 61 and 90 suggests moderate dependence, a total score between 91 and 99 suggests mild dependence, and a total score of 100 suggests independence [24]. The ability to perform instrumental activities of daily living was assessed using the Lawton and Brody scale [25], which assesses eight items (ability to use the telephone, make purchases, prepare food, take care of the house, wash clothes, use of means of transport, self-management of medication, and management of finances). Taking into account gender differences, total dependence was categorized as 0 in men and 0–1 in women, severe dependence was categorized as 1 in men and 2 and 3 in women, moderate dependence was categorized as 2–3 in men and 4–5 in women, mild dependence was categorized as 4 in men and 6–7 in women, and independence was categorized as 5 in men and 8 in women. Ambulation ability was determined using the functional categories of ambulation (FAC) [26], which is a scale with six possible scores (0–5), where a lower score denotes greater dependency. A total score between 0 and 3 indicates that the ambulator is dependent or nonambulatory, whereas a score of 4 or 5 suggests independence. The detection of depressive symptoms in geriatric patients was carried out using the Spanish version of the 15-item Geriatric Depression Scale [27,28]. A score between 0 and 5 indicates no depression, a score between 6 and 9 suggests possible depression, and a score ≥10 reveals established depression. The socio-family situation was determined using the Gijón scale [29], which assesses five dimensions (family situation, economic situation, housing, social relationships, and support from social network). A total score between 5 and 9 indicates a good or adequate social situation, a score of 10–14 indicates social risk, and a score ≥15 indicates a social problem. Comorbidity was calculated using the Charlson comorbidity index [30]. This index is a predictive model that assigns numerical values to different chronic pathologies, obtaining a final score for each individual patient by adding the partial values.

### 2.4. Statistical Analysis

A descriptive analysis of the continuous variables was carried out using the mean (standard deviation), whereas qualitative variables were described with absolute and relative frequencies (%). The analysis of a normal distribution was performed using the Kolmogorov–Smirnov test, and the Levene test was used to test the homoscedasticity. Spearman correlation was used to assess the influence of continuous variables on the quality of life at 12 months.

The experimental groups were compared using the chi-square test, the Student’s *t*-test, or the nonparametric Mann–Whitney and Kruskal–Wallis tests for a non-normal distribution.

Repeated-measures ANOVA was used to analyze score differences in the eight quality-of-life subscales between prefracture and 12 months after surgery.

The variables considered in the analysis of the dimensions (for both the PCS-12 and the MCS-12 summaries, as well as for each of the dimensions) were gender, age, polymedication (yes/no), state of life at the beginning of the study (institutionalized/noninstitutionalized), walking independently (FAC) after 12 months (yes/no), Charlson comorbidity index, the Barthel index, Gijon scale, Yesavage depression scale, and Lawton and Brody scale.

The explanatory variables that showed statistical significance with *p* < 0.10 in the previously performed bivariate analysis were introduced. Statistical significance was considered for *p*-values < 0.05. All analyses were performed with the SPSS 20.0 software (IBM Corp., Chicago, IL, USA).

## 3. Results

**Figure 1:**
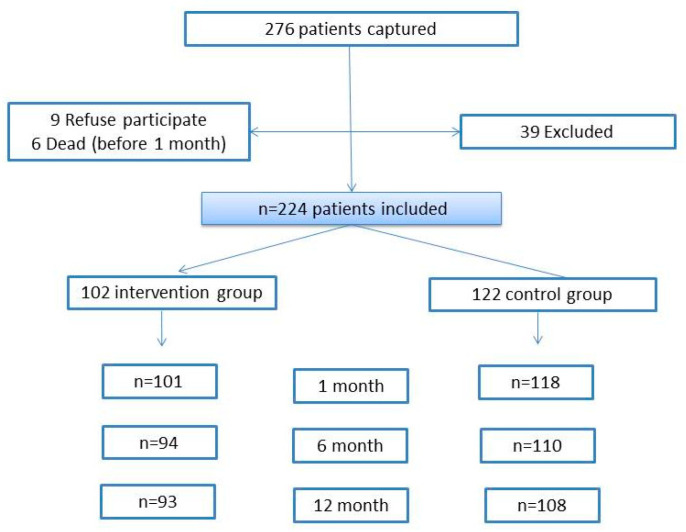
Flowchart of cases.

During the study period, 276 patients were recruited, of which 43 did not meet the inclusion criteria, three refused to participate, and six died before the month’s review. The sample consisted of a total of 224 participants, of which 102 patients (45.5%) received the educational program, while 122 (54.5%) did not (Figure 1).

The baseline demographic and clinical characteristics of the patients are shown in Table 1. No statistically significant differences were found between the two groups. The patients were predominantly women (76.3%), aged 84.6 years (SD 6.1). The mean Charlson comorbidity index at the time of surgery was 5.3 (SD 1.2), and the prevalence of prior diagnosis of osteoporosis was 18.4%. The baseline functional capacity variables (Barthel index, Lawton and Brody scale, and FAC) were not significantly different (*p* = 0.974; *p* = 0.604; *p* = 0.60).

Table 2 shows the evolution of the SF-12 quality-of-life dimensions throughout the study in the intervention and control groups.

The dimensions of vitality and social functioning showed statistically significant differences within groups (*p* = 0.036; *p* = 0.006). That is, there was a significantly better result in the intervention group throughout the study time.

**Figure 2:**
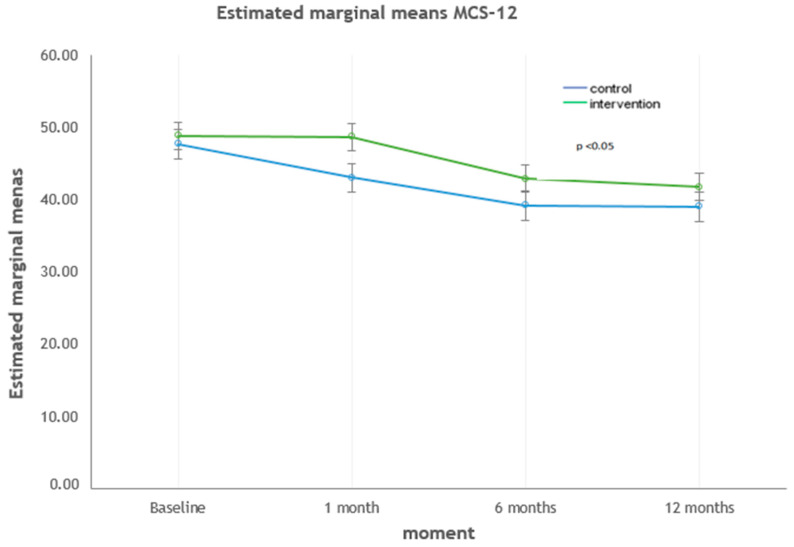
Evolution of the MCS-12 summary dimension: intervention vs. control group.

**Figure 3:**
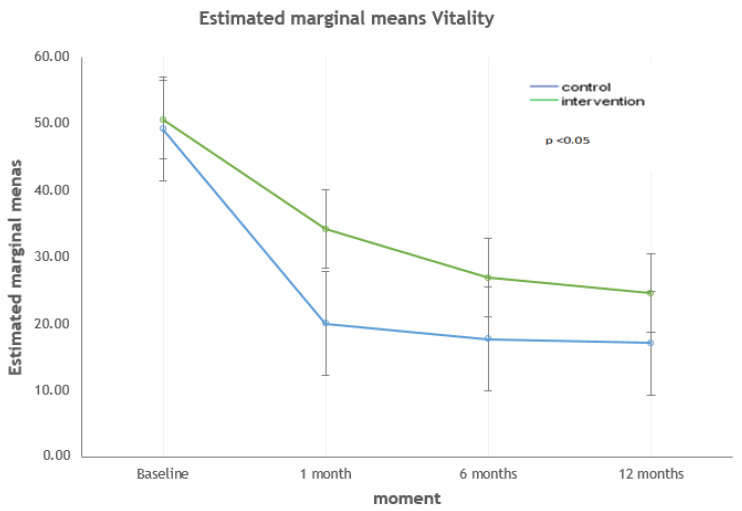
Evolution of the vitality dimension: intervention vs. control group.

**Figure 4:**
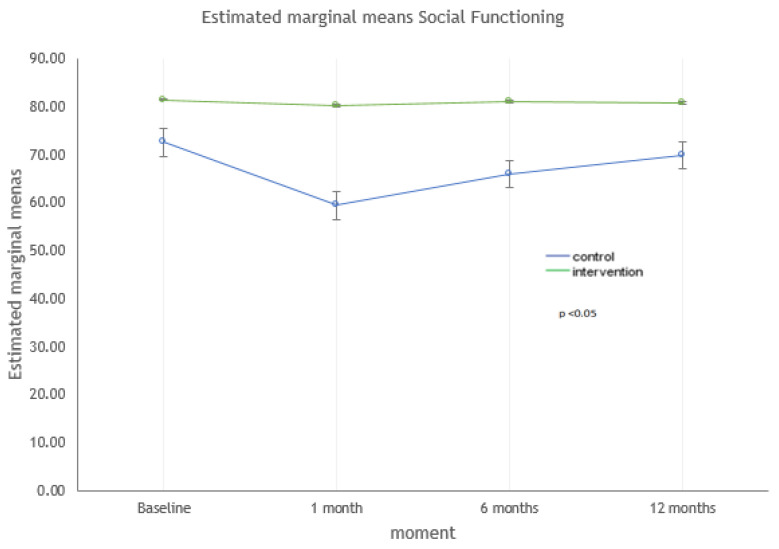
Evolution of the social functioning dimension: intervention vs. control group.

The dimensions that showed statistically significant differences between both groups at 12 months were MCS-12, vitality, and social functioning. The strongest correlations were found with the social functioning dimension (*p* < 0.001). For the MCS-12 dimension (Figure 2), the quality of life was higher in the intervention group (*p* = 0.043). Regarding the dimensions of vitality (Figure 3) and social functioning (Figure 4), their values were higher in the educational intervention group than in the control group (*p* = 0.01 vs. *p* < 0.001), and this difference was maintained over time (*p* = 0.036 vs. *p* = 0.006).

In the univariate analysis (Table 3), we found six parameters that correlated significantly with the MCS-12 summary dimensions at 12 months. These parameters were gender, age, the Barthel index, Gijon scale, Yesavage depression scale, and Lawton and Brody scale. The strongest correlations were found with the Barthel index (Spearman’s rho coefficient = 0.304) and Yesavage depression scale (Spearman’s rho coefficient = −0.562).

The living status, Barthel index, and Lawton and Brody scale correlated significantly with the PCS-12 dimension. The strongest correlations were found with the Barthel index (Spearman’s rho coefficient = 0.265) and Lawton and Brody scale (Spearman’s rho coefficient = 0.290) (Table 3).

Table 4 shows the lineal regression model constructed to assess the predictive factors at 12 months that influenced the eight dimensions and two summary scores of the SF-12 health questionnaire (adjusted *R^2^* = 0.257). At 12 months, the educational intervention variable was associated with higher levels in the dimensions of physical role (B = 4.796 (1.907–7.685); *p* < 0.001), vitality (B = 10.171 (4.505–15.837); *p* < 0.001), and social functioning (B = 10.155 (4.895–15.415); *p* < 0.001).

A higher independence in instrumental activities of daily living at 12 months was associated with higher levels in the dimensions of PCS-12 (B = 1.058, (0.353–1.764); *p* < 0.001), physical function (B = 4.301, (1.607–6.994); *p* < 0.001), physical role (B = 2.369, (1.049–3.689); *p* < 0.001), and vitality (B = 4.825, (2.213–7.438); *p* < 0.001).

The increased ability to perform the basic activities of daily living was associated with higher levels in the physical function (B = 0.286 (0.035–0.536); *p* < 0.001) and general health (B = 0.223, (0.071–0.376); *p* < 0.001) dimensions. However, the Charlson comorbidity index was related to a higher level in the dimension of body pain (B = 2.850, (0.155–5.545); *p* < 0.001).

Age was associated with lower levels in the dimensions of MSC-12 (B = −0.456 (−0.784 to −0.128); *p* < 0.001) and emotional role (B = −0.659, (−1.248 to −0.070); *p* < 0.001). A worse mood (Yesavage depression scale) was associated with lower levels in the dimensions of MSC-12 (B = −1.738, (−2.486 to −0.989); *p* < 0.001), body pain (B = −2.412 (−3.556 to −1.267)); *p* < 0.001), general health (B = −0.969, (−1.651 to −0.286); *p* < 0.001), vitality (B = −1.105, (−2.194 to −0.016); *p* < 0.001), social functioning (B = −1.531, (−2.457 to −0.605); *p* < 0.001), emotional role (B = −3.741, (−5.016 to −2.466); *p* < 0.001), and mental health (B = −2.616, (−3.806 to −1.427), *p* < 0.001).

## 4. Discussion

Rehabilitation after hip fracture surgery is crucial to achieving prefracture functional capacity [31]. The objective of the present study was to verify whether a postoperative intervention program could be useful in patients with hip fracture and, therefore, improve their HRQoL. If so, this educational program could be incorporated into the clinical practice of hip fracture management. To our knowledge, there are few studies correlating these educational programs with HRQoL, and there is little information on the programs and long-term outcomes after surgery. Different studies have evaluated the effect on HRQoL of various educational or counseling interventions focused on specific aspects, such as nutritional status [32] or psychological support [33,34,35], as well as the effect of educational programs framed within a broader rehabilitation strategy [36,37,38,39]; however, very few studies evaluated the impact of an educational program addressing the different preventive and rehabilitative aspects of HRQoL. A study carried out by Cinnella et al. evaluated the short-term impact of an educational program carried out during hospitalization on the HRQoL of 40 patients who underwent operations for hip fracture, using the SF-36 tool. A recent study developed in Spain evaluated the effectiveness of an educational intervention in patients with hip fracture with a 12-month follow-up, although HRQoL was not included in its outcome variables [17].

The population of our study was mainly made up of noninstitutionalized women older than 80 years, in line with other studies aimed at assessing the quality of life in elderly patients with hip fracture [17,34,40,41,42,43,44].

In our study, in both groups, the values of the physical dimension showed a more pronounced decrease 1 month after the intervention, increasing slightly in subsequent evaluations, although far from returning to the baseline values. On the contrary, the values of the mental dimension remained relatively stable during the first month, before decreasing at 6 and 12 months. In a study focused on identifying factors associated with quality of life after 12 months of suffering a hip fracture, [34] showed a progressive decrease in both dimensions during the first 3 months, which was more pronounced in the physical than in the mental dimension. These values were subsequently increased, although the PCS-12 did not recover to its initial values 12 months after the operation, while the MCS-12 did. Along the same lines, in another study carried out on 38 patients where the SF-36 questionnaire was used [45], there was a recovery to the initial values 6 months after the fracture, except for the physical role. Similar to the data found in our study, another study showed little variation at 3 months after surgery in the mental dimension, but wide variation in the physical dimension [46]. In a study carried out in Spain, there was a significant impact on the quality of life of the patient immediately after the fracture, with a partial recovery in the following 4 months, increasing to 60% of that at full health [47]. The study developed by Hallberg et al., with a follow-up period of 2 years, showed a profound impact on HRQoL during the first 6 months and a progressive improvement in HRQoL over time; however, the physical role, physical function, and social function did not return to baseline values [14]. It is expected that, in the first months after surgery, the HRQoL values should experience a significant drop related to the significant decrease in functional development capabilities, and that these values should improve along with functional ability. In our work, the body pain domain was the only one that increased after 12 months until reaching baseline values, even exceeding it in the case of the intervention group. The remaining domains not only did not recover baseline values, but they were far from achieving them, as in the case of the physical function domain, with a recovery of 26.6% in the control group and 25.6% in the intervention group, or in the case of vitality, with a recovery of 34.8% and 48.5%, respectively; in other domains, the degree of recovery was around 60%.

In the study by Cinnella et al., counseling had a positive impact on the quality of life of all patients, but was more relevant to patients who had low HRQoL scores at the time of admission [18]. Taking into account the domains of each dimension, they found a significant association of counseling with the physical function, physical role, emotional role, mental health, and vitality. In our study, we found a significant association between the educational session and vitality and social functioning, with the latter understood as the degree of physical and emotional health affecting habitual social life (both components of the mental dimension), although we did not find an association between the educational program and the physical dimension. A study aimed at comparing the short-term effects on fear of falling from a physical activity program versus an educational program showed that gains in the perception of health status were limited to physical health for the activity group and mental health for the education group [48], which could explain why the educational intervention proposed in this study had a certain influence on the mental dimension and not the physical one.

Regarding the factors with a direct influence on HRQoL, different systematic reviews identified a low physical level or psychosocial functioning before the hip fracture, comorbidity, female gender, poor nutritional status, unstable extracapsular fractures, perception of severe postoperative pain, longer length of hospital stay, and postoperative complications to be associated with lower HRQoL values [11,16]. Our study showed a significant association of an improvement in the physical dimension with living together before the fracture and with the development of basic and instrumental daily activities at 12 months. On the contrary, the association was negative with social risk. In the study developed by Moreman et al. [34], the decrease in values in the physical dimension was associated with a higher level of mobility before the fracture, intracapsular fracture, treatment with osteosynthesis, and a duration of stay greater than 9 days, with a greater recovery of HRQoL in terms of the PCS-12 between 3 and 12 months; in this study, having a partner at admission was associated with better PCS-12 scores at admission, but not in subsequent measurements.

In the case of the mental dimension, we found that it was positively influenced by the female sex and the development of basic and instrumental activities, but negatively influenced by age, social risk, and the presence of depression.

In a study aimed at determining the prognostic factors of perceived health 3 months after surgery, the use of walking aids before the fracture was evidenced as a significant and independent risk factor for a low PCS-12 score, PCS-12 score prefracture, and cognitive dysfunction, while the only statistically significant and independent risk factor for a low MCS-12 score was the prefracture MCS-12 score, and while the female gender demonstrated marginal significance [46].

In our study, the effectiveness of the educational program was confirmed in our patients from the results obtained in the mental health dimension, whereby the application of a structured educational program developed during hospitalization could influence the psychosocial conditions and self-perceptions of the patient throughout the recovery process, thereby improving self-care and obtaining a better HRQoL. More research is needed in this regard to delimit the direct influence of educational programs on the quality of life of patients and to determine what types of patients could benefit from such an intervention.

### Limitations

A limitation of the study stems from the impossibility of obtaining information prospectively on the situation prior to the fracture, thereby leading to the possibility of memory bias and underestimation of the results [49]. In addition, the measurement of HRQoL at 12 months could have been influenced by factors not related to the hip fracture, but to pre-existing comorbidities [50], which is an aspect of special relevance when taking into account the advanced age and comorbidity of our study population. Although we are aware of this limitation, it is intrinsically linked to studies aimed at evaluating the impact of certain pathologies on HRQoL. Our reference point was, in accordance with most studies, the baseline situation referred to two weeks before the fracture, such that, if present, this memory bias would be minimal, since the survey was carried out during hospital admission. Furthermore, our multivariate analysis ruled out comorbidities (Charlson index) on admission as an independent factor associated with HRQoL. Another limitation was that the NCT registration was not performed at the beginning of the study.

We also recognize the lack of blinding to the health education program as a weakness in our study.

## 5. Conclusions

During one year of follow-up, hip fracture significantly affected the quality of life in the patients studied. The intervention group showed significant improvements in the MCS-12, vitality, and social functioning dimensions with respect to the control group.

Further studies are needed to clarify the direct influence of the mental domain on functional recovery in older patients affected by hip fracture. Identifying these patients early could be of benefit to the implementation of educational programs.

## Figures and Tables

**Table 1:** Baseline demographic and clinical characteristics of patients. ASA PS, American Society of Anesthesiologists physical state.

	Intervention Group (*n* = 102, 45.5%)	Control Group (*n* = 122, 54.5%)	*p*-Value *
Gender, *n* (%)			
Female	80 (78.4)	91 (74.6)	0.501
Male	22 (21.6)	31 (25.4)	
Age, mean (SD) ^$^	84.4 (5.5)	84.8 (6.6)	0.320
Groups, *n* (%)			0.070
<85 years	55 (53.9)	51 (41.8)	
≥85 years	47 (46.1)	71 (58.2)	
Study level, *n* (%)			0.254
No studies	45 (44.1)	40 (32.8)	
Primary	52 (51.0)	78 (63.9)	
Secondary	3 (2.9)	3 (2.5)	
University	2 (2.0)	1 (0.8)	
Living status, *n* (%)			0.552
Living alone	24 (23.5)	33 (27.0)	
Living in couple	26 (25.5)	35 (28.7)	
Living with relatives	34 (33.3)	28 (23.0)	
Supervised flat	3 (2.9)	4 (3.3)	
Residency	15 (14.7)	22 (18.0)	
Clinical history, *n* (%)			
Prior diagnosis of Osteoporosis	7 (6.9)	14 (11.5)	0.238
Previous hip fracture, *n* (%)	11 (10.8)	7 (5.7)	0.166
Polymedication, *n* (%)	78 (76.5)	78 (63.9)	0.154
Charlson comorbidity index, mean (SD)	5.2 (1.0)	5.4 (1.3)	0.421
Type of fracture, *n* (%)			0.079
Neck	36 (35.3)	44 (36.1)	
Trochanter	66 (64.7)	78 (63.9)	
Type of surgical intervention, *n* (%)			0.749
Intramedullary nail	68 (67.3)	79 (65.3)	
Hip replacement	33 (32.7)	42 (34.7)	
ASA PS for peri-operative risk, *n* (%)			0.075
I	1 (1.0)	0 (0.0)	
II	21 (20.6)	49 (40.2)	
III	70 (68.6)	66 (54.1)	
IV	10 (9.8)	7 (5.7)	
Destination after surgery, *n* (%)			0.392
Home	49 (48.0)	49 (40.2)	
Institution	33 (31.4)	52 (42.6)	
With relatives	20 (19.6)	20 (16.4)	
Barthel index, mean (SD)	88.18 (16.22)	87.09 (17.47)	0.974
Lawton and Brody scale, mean (SD)	5.20 (2.59)	4.93 (2.75)	0.604
Functional Ambulation Classification (FAC): Does not walk independently, *n* (%)	33 (32.7%)	62 (51.2%)	0.060

* Pearson χ^2^; ^$^ Mann–Whitney U.

**Table 2:** Quality-of-life dimensions of the SF-12 health survey throughout the study: intervention vs. control group. MCS-12, Mental Component Summary; PCS-12, Physical Component Summary.

	Baseline		1 Month		6 Months		12 Months		Within (Intra)	Between (Inter)
	Mean (SD)		Mean (SD)		Mean (SD)		Mean (SD)		*p*-Value ^#^	*p*-Value ^#^
	Control	Intervention	Control	Intervention	Control	Intervention	Control	Intervention		
	62	73	62	73	62	73	62	73		
MCS-12	47.7 (10.8)	48.9 (11,1)	43 (13.2)	48.7 (12.2)	39.3 (11.8)	42.9 (11.7)	39.1 (12.1)	41.8 (11.4)	0.177	0.043
PCS-12	39.9 (10.9)	39.4 (11.4)	29.0 (5.4)	28.5 (4.5)	32.5 (6.4)	31.4 (4.8)	32.8 (5.9)	32.4 (5.7)	0.092	0.460
Physical function	50.3 (39.4)	51.9 (41.5)	4.5 (17.1)	3.7 (13.2)	13.7 (24.4)	11.2 (23.8)	13.4 (24.1)	13.3 (28.4)	0.741	0.880
Physical role	51.3 (24.6)	49.8 (24.9)	25.5 (5.1)	25.5 (3.4)	28.4 (11.9)	28.3 (12.4)	26.1 (6.2)	30.1 (15.3)	0.228	0.645
Bodily pain	68.9 (28.4)	64.2 (32.8)	55.5 (22.8)	59.1 (24.1)	66.6 (20.5)	67.3 (24.1)	68.4 (22.5)	69.2 (23.4)	0.253	0.973
General health	46.3 (21.6)	50.7 (21.3)	32.1 (16.2)	32.7 (16.9)	28.4 (14.4)	29.4 (17.4)	27.9 (15.4)	26.9 (17.7)	0.195	0.530
Vitality	49.1 (33.3)	50.5 (30.8)	20.0 (24.9)	34.1 (28.6)	17.6 (20.3)	26.8 (25.4)	17.1 (21.9)	24.5 (25.9)	0.036	0.010
Social functioning	72.6 (24.7)	81,5 (24.8)	59.5 (28.1)	80.4 (26.2)	66.1 (22.7)	81.1 (23.3)	70.0 (21.1)	80.8 (20.2)	0.006	<0.001
Emotional role	64.7 (19.5)	61.9 (21.8)	52.4 (24.7)	57.0 (23.9)	46.1 (24.5)	47.4 (24.9)	44.7 (24.4)	45.6 (24.7)	0.217	0.694
Mental health	65.4 (23.7)	64.5 (24.2)	49.3 (22.2)	58.2 (22.6)	44.7 (19.7)	48.6 (20.8)	42.4 (20.7)	44.4 (20.2)	0.056	0.224

^#^ Repeated-measures ANOVA.

**Table 3:** Variables associated with the summary dimensions of the SF-12 questionnaire at 12 months after the surgical intervention.

	**Physical Component Summary (PCS-12) 12 Months**		**Mental Component Summary (MCS-12) 12 Months**	
	**Mean (SD)**	***p*-Value ^$^**	**Mean (SD)**	***p*-Value ^$^**
Gender	31.6 (6.2)	0.094	46.7 (13)	0.006
Polymedication	38 (33.8)	0.135	38 (42.9)	0.372
Living status	68 (33.3)	0.023	68 (41.4)	0.575
Functional Ambulation Classification (FAC)	77 (32.3)	0.626	77 (39)	0.050
	**Rho**	***p*-Value ^&^**	**Rho**	***p*-Value ^&^**
Age	0.026	0.727	−0.216	0.003
Charlson comorbidity index	0.049	0.502	−0.119	0.105
Barthel index 12 months	0.265	<0.001	0.304	<0.001
Gijon scale 12 months	−0.143	0.050	−0.194	0.008
Yesavage depression scale 12 months	−0.075	0.305	−0.562	<0.001
Lawton and Brody scale 12 months	0.290	<0.001	0.211	0.004

^$^ Mann–Whitney U; ^&^ Spearman correlation coefficient.

**Table 4:** Multiple regression analysis of factors influencing dimensions of the SF-12 questionnaire at 12 months after the surgical intervention.

						MODEL		
		B	IC _95%_ to B		Sig.	*R^2^*	*R^2^*_c_	*p*-Value
Physical Component Summary (PCS-12)	Lawton and Brody scale	1.058	0.353	1.764	0.003	0.203	0.176	<0.001
Mental Component Summary (MCS-12)	Age	−0.456	−0.784	−0.128	0.007	0.357	0.314	<0.001
	Yesavage depression scale	−1.738	−2.486	−0.989	<0.001			
Physical function	Barthel index	0.286	0.035	0.536	0.026	0.412	0.378	<0.001
	Lawton and Brody scale	4.301	1.607	6.994	0.002			
Physical role	Intervention	4.796	1.907	7.685	0.001	0.304	0.268	<0.001
	Lawton and Brody scale	2.369	1.049	3.689	0.001			
Bodily pain	Charlson comorbidity index	2.850	0.155	5.545	0.038			
	Yesavage depression scale	−2.412	−3.556	−1.267	<0.001			
General health	Barthel index	0.223	0.071	0.376	0.004			
	Yesavage depression scale	−0.969	−1.651	−0.286	0.006			
Vitality	Intervention	10.171	4.505	15.837	0.001	0.413	0.379	<0.001
	Yesavage geriatric depression scale	−1.105	−2.194	−0.016	0.047			
	Lawton and Brody scale	4.825	2.213	7.438	0.000			
Social functioning	Intervention	10.155	4.895	15.415	<0.001	0.267	0.249	<0.001
	Yesavage depression scale	−1.531	−2.457	−0.605	0.001			
Emotional role	Age	−0.659	−1.248	−0.070	0.029	0.330	0.294	<0.001
	Yesavage depression scale	−3.741	−5.016	−2.466	<0.001			
Mental health	Yesavage depression scale	−2.616	−3.806	−1.427	<0.001	0.323	0.281	<0.001

## Abbreviations

ASA	American Society of Anesthesiologists
BI	Barthel index
CCI	Charlson comorbidity index
HRQoL	Health-related quality of life
FAC	Functional Ambulation Classification
MCS-12	Mental summary dimension
PCS-12	Physical summary dimension
SF-12	SF-12 Health Survey
SD	Standard deviation
